# Electronic reporting of integrated disease surveillance and response: lessons learned from northeast, Nigeria, 2019

**DOI:** 10.1186/s12889-021-10957-9

**Published:** 2021-05-13

**Authors:** Luka Mangveep Ibrahim, Ifeanyi Okudo, Mary Stephen, Opeayo Ogundiran, Jerry Shitta Pantuvo, Daniel Rasheed Oyaole, Sisay Gashu Tegegne, Abdelrahim Khalid, Elsie Ilori, Olubunmi Ojo, Chikwe Ihekweazu, Fiona Baraka, Walter Kazadi Mulombo, Clement Lugala Peter Lasuba, Peter Nsubuga, Wondimagegnehu Alemu

**Affiliations:** 1 World Health Organization, Rivers House, #83 Ralph Shodeinde Street, Abuja, Nigeria; 2 WHO Africa Regional Office, Brazzaville, Congo; 3 Nigerian Center for Disease Control, Jabi, Abuja, Nigeria; 4 Global Public Health Solutions, Atlanta, GA USA; 5 International Health Consultancy, Atlanta, GA USA

**Keywords:** Integrated disease surveillance and response, Electronic reporting, eIDSR, Nigeria

## Abstract

**Background:**

Electronic reporting of integrated disease surveillance and response (eIDSR) was implemented in Adamawa and Yobe states, Northeastern Nigeria, as an innovative strategy to improve disease reporting. Its objectives were to improve the timeliness and completeness of IDSR reporting by health facilities, prompt identification of public health events, timely information sharing, and public health action. We evaluated the project to determine whether it met its set objectives.

**Method:**

We conducted a cross-sectional study to assess and document the lessons learned from the project. We reviewed the performance of the local government areas (LGAs) on timeliness and completeness of reporting, rumors identification, and reporting on the eIDSR and the traditional paper-based system using a checklist. Respondents were interviewed online on the relevance, efficiency, sustainability, project progress and effectiveness, the effectiveness of management, and potential impact and scalability of the strategy using structured questionnaires. Data were cleaned, analyzed, and presented as proportions using an MS Excel spreadsheet. Responses were also presented as direct quotes.

**Results:**

The number of health facilities reporting IDSR increased from 103 to 228 (117%) before and after implementation of the eIDSR respectively. The timeliness of reporting was 43% in the LGA compared to 73% in health facilities implementing eIDSR. The completeness of IDSR reports in the last 6 months before the evaluation was ≥85%. Of the 201 rumors identified and verified, 161 (80%) were from the eIDSR pilot sites. The majority of the stakeholders interviewed believed that eIDSR met its predetermined objectives for public health surveillance. The benefits of eIDSR included timely reporting and response to alerts and disease outbreaks, improved timeliness, and completeness of reporting, and supportive supervision to the operational levels. The strategy helped stakeholders to appreciate their roles in public health surveillance.

**Conclusion:**

The eIDSR has increased the number of health facilities reporting IDSR, enabled early identification, reporting, and verification of alerts, improved timeliness and completeness of reports, and supportive supervision of staff at the operational levels. It was well accepted by the stakeholder as a system that made reporting easy with the potential to improve the public health surveillance system in Nigeria.

**Supplementary Information:**

The online version contains supplementary material available at 10.1186/s12889-021-10957-9.

## Background

Public health surveillance remains the cornerstone to overcome health threats affecting humans and their environments globally. The functionality of a surveillance system is gauged by its capacity to prevent, detect and report the outbreaks of disease, conditions, and events when they occur on time, and promptly respond to contain and control the outbreaks [1–3]. An efficient surveillance system is also required to monitor and measure the impact of public health interventions. Its success depends on a robust information system with reliable and timely data collection, collation, analysis, interpretation, and transmission of the information for action.

The Integrated Disease Surveillance and Response (IDSR) is the adopted strategy for public health surveillance in Nigeria as with other member states in the African sub-region of the World Health Organization (WHO). According to the WHO, strategy provides a rational basis for decision-making and implementation of public health interventions that are efficacious in responding to priority diseases, conditions, and events [4]. It was adopted in Nigeria in 2001 to improve the ability of local government areas (LGAs) to detect and promptly respond to outbreaks of diseases, conditions, and events that have the potential to cause high levels of mortality, morbidity, and disabilities [5]. The health facility (primary, secondary, or tertiary) is the basic operational unit and the primary point for the generation of the IDSR data. At this level, a surveillance focal person extracts data on the priority diseases from the in-patient or out-patient registers of the health facility into the IDSR reporting form. The completed forms are sent to the Local Government Area Disease Surveillance and Notification Officer (LGA DSNO). The LGA DSNO collates the reports from all health facilities in the LGA into a single form for transmission to the State DSNO and State Epidemiologist. The process of extracting the data from the health facilities to the IDSR forms is manual and paper-based. Reports are transmitted physically by the surveillance focal persons from the health facility to the LGA DSNO. The manual extraction and physical transmission are usually cumbersome and the risk of missing important information delayed transmission of information for action and conversely delayed response especially if it an infectious disease [6]. The delays in detection and reporting of diseases such as Lassa fever, measles, cerebrospinal meningitis, and Yellow fever resulting in outbreaks in the country [7–11]. These outbreaks highlight the problems with the traditional way of implementing the IDSR strategy in the country [12, 13]. The speed of information transmission is one of the important qualities of public health surveillance system to ensure prompt public health actions to limit the spread of outbreaks caused by infectious diseases [14–16]. The application of information technology (IT) in public health surveillance facilitates early detection and reporting of disease outbreaks including tracking of response to public health threats. The system eliminates the manual extraction of surveillance from the source document thereby improving the quality and reliability of the data [17–19]. Some countries have utilized the technology in their public health surveillance systems including IDSR. In Africa, Sierra Leone is one of the first countries that had fully digitalized the IDSR reporting system at all levels of the health system with promising outcomes [20, 21]. In Nigeria, an electronic system, the mobile Strengthening Emergency and Response System (mSERS) is being used to transmit the weekly reports by the LGA DSNO. The system had not improved early detection and reporting of surveillance data from the health facilities because it is stationed at the level of the LGA DSNO and relies on the manual extraction and physical transmission of the data from the reporting sites [19, 22].

There is a need for a system that will enhance the performance of disease surveillance and response particularly with early reporting from the health facility level in the country. An electronic system that captures IDSR data from the health facilities was considered a better approach to improve the public health surveillance system in the country. The idea was perceived as part of the revitalization of the disease surveillance and response system and drawn from the experience of application of the early warning alert and response system (EWARS) in security challenged areas (Borno state) in the country. The goal of the electronic reporting of the IDSR data to strengthen the disease surveillance system for early detection and real-time reporting enabling prompt response to outbreaks including rumor verification and reporting.

The objectives were to:

- build capacity of health facility personnel, the LGA, and State on detection, reporting, and response to outbreaks of diseases and public health events in the country
- build the capacity of the LGA and state DSNOs, the state Epidemiologists, and national staff on the coordination roles for disease surveillance and management of the surveillance data including the provision of supportive supervision to the lower levels
- improve on the quality of the surveillance data for evidence-based decision-making.
- ensure real-time data reporting from the health facility for prompt action

The project was implemented in 10 LGAs from two states in North-East Nigeria. The implementation had a two-phased approach; the first phase was to strengthen the IDSR reporting system at the health facilities, LGAs, and state levels. These included the provision of standard case definitions of the priority diseases under surveillance in the country, IDSR reporting tools, and training of the State, LGA and health facility surveillance focal persons on the reporting system. This approach was based on the premise that introducing a new concept or innovation in a weak system would be worthless. The electronic reporting of the IDSR data can only function in a system where the traditional system is already working [23].

The second phase was the introduction of the eIDSR in the selected health facilities. A total of 54 health facilities from 10 LGAs drawn from two states were selected for the initial implementation. The selection criteria were; location of the health facilities, participation of the health facility in IDSR reporting, availability of the mobile network, accessibility, and security concerns. An application was developed for the eIDSR by a team of specialists who had worked on a previously successful electronic application for the Auto-visual AFP detection and response (AVADAR) system in the polio eradication initiative project. The IDSR immediate notification, weekly and monthly reporting forms, as well as the supervision checklist, were converted into electronic format. A task team was formed to develop a blueprint and to coordinate the implementation of the eIDSR. The members of the task team were also trained on the application which was subsequently field-tested by the team. Surveillance focal persons and officers in charge of the selected health facilities were trained on the use of mobile phones to collect and report IDSR data. A total of 108 staff from 10 health facilities in the two states were selected to pilot the eIDSR. A supervision plan was also developed for the eIDSR. In the plan, the supervisors from states and LGAs were to conduct supportive supervision on the facilities at least once a week using a checklist. Monthly and quarterly meetings were to be held at the state and national levels respectively to review the progress of implementation of the project, address challenges, and proffer solutions.

We evaluated the initial implementation of the eIDSR to determine whether the project met its predetermined objectives for improving timeliness and completeness of IDSR reporting, prompt identification of public health events, timely information sharing, and use of the system by the key players in disease surveillance in the selected health facilities and LGAs for actions.

## Methods

### Study design

We conducted a cross-sectional study to assess and document the lessons learned from the initial implementation of the eIDSR. We reviewed and extracted data on some of the key performance indicators for IDSR implementation at the LGA levels using checklists and interviewed surveillance officers and clinicians on eIDSR using structured questionnaires sent to their mobile phones. Participants were selected from the health facilities implementing the eIDSR. Each respondent that consented to participate in the assessment completed the questionnaire and submitted it online. The questionnaires were adapted from a set of tools for the evaluation of public surveillance systems. Separate tools were developed for respondents at the health facilities and the stakeholders at the LGA and state levels [24, 25].

### Study setting

Nigeria has a federal system of government made up of 36 states and a Federal Capital Territory (FCT) with 774 constitutionally recognized local government areas. Yobe and Adamawa state, our study areas are located in the northeastern part of the country. They have an estimated population of 5.5 million people extrapolated from the 2006 national census [26]. The evaluation was conducted in 54 health facilities from 10 LGAs involved in the eIDSR project.

### Data collection

We conducted a desk review of the performance of the LGAs on the reporting of IDSR data. The review included the number of health facilities reporting IDSR before and after the introduction of the eIDSR; the number of rumors identified by the eIDSR system compared to the traditional system; timelines and completeness of weekly IDSR reporting from health facilities implementing eIDSR compared to the traditional system using checklists. The Surveillance focal persons and clinician working at the piloting health facilities, LGA and State DSNO, and State Epidemiologist were interviewed online using structured questionnaires on the following six core theme for evaluation of a surveillance system:

- **The relevance of the strategy**: questions were asked on the extent to which the activities designed and implemented were suited to the priorities and realities of the Nigerian context.
- **Project Progress and Effectiveness**: To explore the extent to which the program has adequately achieved its intended outputs and objectives such as prompt identification of public health threats, facilitation of data collection, validation and real-time analysis of data, provisions of a platform for efficient information management and timely information sharing with stakeholders, and generation of accurate weekly aggregate reports.
- **Sustainability**: To assess the ability of supported activities and functions to continue after the project ends.
- **Effectiveness of Management Arrangements**: To explore the extent to which the system brought together relevant stakeholders to achieve project objectives.
- **Potential Impact and Scalability**: To assess the likelihood and extent to which the project will contribute to longer-term improvements in the electronic disease early warning system and scale up to the remaining health facilities in the two states in Nigeria.

### Data analysis

Data from the desk review and online evaluation were entered into an MS Excel spreadsheet cleaned, analyzed, and presented as proportions. Responses were also presented as direct quotes.

### Ethical considerations

We obtained ethical clearance for the study from the National Health Research Ethics Committee of Nigeria (NHREC) in the Department of Planning Research and Statistic of the Federal Ministry of Health Nigeria, reference number NHREC/01/01/2007–03/03/2020. Informed written consent was also obtained from all respondents involved in the study.

## Results

**Fig. 1:**
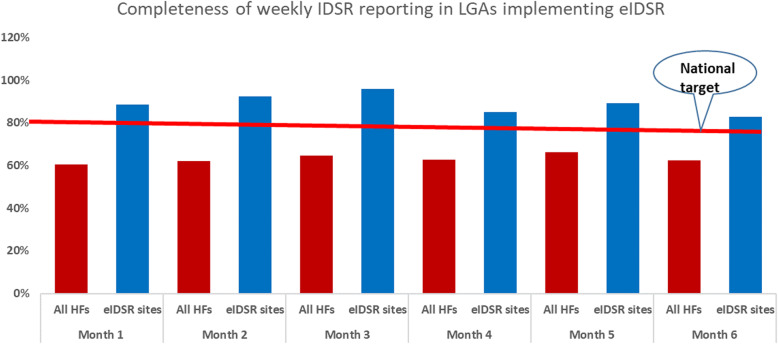
Completeness of weekly IDSR reporting in all and health facilities with eIDSR Legend:

A total of 54 health facilities were involved in the eIDSR pilot, of which 45 (87%) participated in the evaluation. Forty (89%) were public, and 5(11%) were private health facilities. The number of health facilities reporting IDSR in the 10 LGAs increased from 103 to 228 (121%) before and after the implementation of eIDSR respectively. The average timeliness of reporting in the last 6 months before the evaluation was only 43% in the piloting LGAs compared to 73% in health facilities implementing eIDSR. The completeness of reports was ≥85% in the health facilities with eIDSR compared to ≤65% in the remaining health facilities in the LGAs (Fig. 1). Also, of the 201 rumors identified and verified in the 6 months before the evaluation, 161 (80%) were from health facilities implementing eIDSR. A total of 45 staff at the health facilities and 21 stakeholders at the LGA levels responded to the online questionnaire. The respondents at the health facilities were 23 (51%) surveillance focal persons, 13 (29%) officers in charge of the health facilities, 7 (16%) Assistant DSNOs, and 2 (4%) Local government area facilitators (LGAFs). Furthermore, of the 21 stakeholders, 11 (52%) were DSNOs, 5 (24%) were WHO local government facilitators (WHO LGAF), 3 (14%) were WHO Cluster coordinators, and 2 (10%) were AVADAR coordinators.

The respondents reported benefits for surveillance using eIDSR to include timely reporting and prompt response to alert and outbreaks of diseases. The majority of the stakeholders believed that eIDSR met the requirement of public health surveillance (Table 1). Similarly, all the respondents in the pilot health facilities had received supportive supervision. A third of the respondents asserted that the eIDSR allowed for analysis of the surveillance data at the local level, 42% mentioned that eIDSR implementation was an added burden to their routine work, and more than a third (38%) could not send reports of alerts within 2 h of detection. The perceived central role of the health facility staff in the design of the eIDSR in Nigeria was reporting diseases to a higher level (Table 2).

**Table 1:** eIDSR surveillance attributes from stakeholders’ viewpoint (*n* = 21)

Attributes	Frequency Yes (%)
Do users find eIDSR useful?	20 (95.2)
Do users find eIDSR simple to use?	20 (95.2)
Is eIDSR a cost-effective option for Nigeria surveillance system	20 (95.2)
Do users find eIDSR acceptable?	18 (85.7)
Is eIDSR sensitive to identify public health problems at the health facility level?	21 (100)
Is eIDSR representative of all public health problems at the health facility level?	18 (85.7)
Does eIDSR provide timely notifications?	21 (100)
Is eIDSR stable (or reliable)?	18 (85.7)
Does eIDSR provide quality data for decision-making?	20 (95.2)
Is eIDSR flexible (can other diseases be reported through it)?	19 (90.5)

**Table 2:** Views of respondents on the design and implementation of eIDSR

Theme	Queries	Yes (%)	No (%)	Total
The relevance of the eIDSR	eIDSR designed according to Nigerian Context	45 (100)	0 (0)	45
	Staff trained before implementation of eIDSR	44 (98)	1 (2)	45
	Staff received supportive supervision during implementation of eIDSR	42 (93)	3 (7)	45
	eIDSR suitable for health facilities reporting	20 (95)	1 (5)	21
Project progress and effectiveness	Alert detected in the last 3 months by the system	25 (56)	20 (44)	45
	The time lag of 2 h or less between detection and reporting of alerts	28 (62)	17 (38)	45
	eIDSR data analyzed at the local levels	7 (33)	14 (67)	21
	eIDSR used for action at the health facility levels	44 (98)	1 (2)	45
	Implementation of eIDSR added burden to the work of the staff	19 (42)	26 (58)	45
	eIDSR is a cost-effective option for public health surveillance in Nigeria	20 (95)	1 (5)	21
Efficiency	eIDSR reflects the efforts staff put into the surveillance system	16 (76)	5 (24)	21
	The system will contribute to e-surveillance in the long term	21 (100)	0 (0)	21

Deductions from the respondents on the project were as follows;

- eIDSR has demonstrated the need and feasibility for an electronic solution for event-based and indicator-based surveillance and response in Nigeria, which is the basis for eSurveillance.
- It was easy to use at the health facility level.
- eIDSR provided regular data updates to higher levels.
- eIDSR was capable of sending alerts within 2 h of detection of public health events. and had been used to send alerts from health facilities in several instances over the past 3 months.
- eIDSR was found to be useful at all levels, particularly in providing timely alerts of public health conditions and events for prompt investigation.
- eIDSR had demonstrated the importance of mobile technology in event-based reporting (Table 3).

**Table 3:** Some direct quotes from respondents on the design and implementation of the eIDSR

Theme	Comments/quotes from respondents
DSNO/State epidemiologists views on the extent to which eIDSR achieved its objectives	It improves the knowledge gap of health workers, through training, supervision, and on-the-job training.
	Immediate notifications and actions were taken on diseases and conditions of public health concerns at the grassroots level.
	It improved the disease surveillance system, increase the flow of data, and improve the early detection and investigation of diseases and conditions of public health concerns.
Surveillance focal person views on the major achievements of eIDSR in Nigeria	It helped in the instant notification of cases that led to the investigation and appropriate public health interventions. More so, it brought line managers closer to surveillance happening at the peripheral level.
	The major achievements included the illustration of how mobile technology can be used to report alerts of IDSR cases, data collection can occur at facilities and be readily available at all level, and dashboards for the ministry of health
	It can be used to show data in real-time, and alerts can be generated to inform the leadership of potential disease outbreaks.
DSNO/State epidemiologists views on the ability to roll out eIDSR	It helps in reduction in the printing of data tools
	It reduces the cost of paper or written materials to do the job.
	Surveillance data will be easily accessed with eIDSR than the traditional method of reporting. Data reported through the traditional system can be altered along the channel of reporting due to manual compilation
Surveillance focal person views on how using eIDSR have benefited their state and Nigeria	It has contributed a lot in identifying and reporting priority diseases and other conditions of public health concern to the responsible authorities, for prompt intervention
	eIDSR contributed to public health surveillance and response in the community
	It makes it easier to report priority diseases timely and completely for prompt action
DSNO/State epidemiologists view on the output of eIDSR relative to the effort put in it	It improved timely reporting and eliminate missing reports
	It keeps the LGA and state informed about immediately reportable diseases
	The system prompted me to verify cases from sources before sending them to a higher level for action
	The system helped me to participate more actively in surveillance activities in my LGA
How eIDSR could contribute to public health surveillance in Nigeria	It helped to improved reporting and response.
	eIDSR has made reporting easier. Therefore, in the future, it will contribute greatly to surveillance such that diseases will be reported immediately for action.
	It will help the country report on time outbreaks and other conditions of public health concerns in the communities. It also helped prompt the detection of cases that came from the community.

## Discussion

The evaluation of the eIDSR implementation revealed that the strategy contributed significantly to improving the operation of the integrated disease surveillance and response in the states. The major contributions were on the numerical increase in health facilities reporting and some of the critical key performance indicators for IDSR. The increase in health facilities reporting IDSR in the selected LGAs might have been due to the availability of the tools to support the implementation. The support tools included the case definitions for the priority diseases, all the reporting forms to all health facilities in the selected LGAs. Another factor that might have contributed to the observed improvement is the refresher training to the surveillance officers at the state and LGA levels. The training built the capacity of the surveillance officers at the LGA and the State to be better prepared to support the surveillance focal persons in the health facilities through supportive supervision, regular feedbacks, verification of alerts, and provision of IDSR data collection and reporting tools.

The importance of quality training in successfully implementing a public health surveillance using the information technology was reported by Njeru et al. from Kenya [27]. Similarly, experiences shared from Uganda among participants at a focus group discussion also showed that training on IDSR at operational levels led to improvement in the completeness and timeliness of reporting, detection of diseases of public health importance, and use of data for decision making [28, 29]. Therefore, strengthening the existing IDSR system is essential consideration before the introduction of eIDSR. Timeliness and completeness of reporting are two critical performance indicators of a surveillance system. Timely reporting of diseases especially communicable diseases is critical in the early detection of outbreaks and presents an opportunity for reducing morbidity, mortality and disabilities associated with the outbreak. In the traditional paper-based method of transmission of reports, reports can be delayed or lost in transit. Electronic reporting increase the speed of transmission, ensure the quality and reliability of the data generated from the reporting sites [30].

The results from our project are supported by the finding of Rebecca Wurtz and Bruce J. Cameron on electronic laboratory reporting (ELR). In their report, ELR increased the speed of completeness of the reporting [31, 32]. Our pilot project revealed an increase in timeliness and completeness which are some of the key performance indicators of the IDSR commonly assessed at the health facility, LGA, and state levels in Nigeria [23, 33]. The role of electronic reporting in improving timeliness and completeness is also corroborated by Randriamiarana R et al. from Madagascar [34], who noted that short message services (SMS) improved the completeness and quality of IDSR data. An electronic reporting system that captures data at the reporting sites is proven to be more efficient and effective in the reporting of the IDSR data. The result also revealed that the electronic transmission of data from health facilities improved the detection, reporting, and verification of alerts.

According to the health workers that responded to our survey the newly introduced eIDSR improved their work. However, a significant proportion (42%) were of the view that the system was an added burden to their routine work. The added burden averred by the respondents could have been due to inadequate manpower in some of the rural health facilities. Soto G et al., who evaluated a four-year implementation of an electronic disease surveillance system in a resource-limited setting reported similar challenges. Other challenges of the electronic surveillance system reported by the authors included lack of phones and limited access to internet services [35]. The lack of mobile phones was not a challenge of implementation of the eIDSR in our study. Mobile phones were given to the focal persons of all participating health facilities. The fact that the focal persons could use the phones as their private phones as against only transmitting the IDSR reports could have contributed to the observed variance. The results of our pilot also showed that there was an improvement in the level of supportive supervision to the operational level. Supportive supervision was identified as one of the critical success factors for the project. It helped to sustain good quality services, identify problems, decide what has caused the problem, and develop feasible solutions. The training provided to all the supervisors on the concept of supportive supervision might have contributed to improving performance of the supervisors. Furthermore, the use of a mobile application, the ODK to collect the geo-coordinate of the health facility, time spent in each health facility supervised and real-time transmission of the supervision report might have contributed to their commitments.

The evaluation had some limitations. Firstly, all the respondents were participants in the pilot. Their views could have been influenced by their role in the project and biased to demonstrate its successes. However, the investigation team adjusted for this bias by analyzing the responses based on a predetermined contextual framework. Secondly, we envisage recall bias as one of the major limitations because the evaluation questions required the respondents to have adequate recall of events that occurred in the past. However, we tried to triangulate sources of information and reduced the recall period to limit the effect of the recall bias.

## Conclusion

The evaluation of the eIDSR project in the two states has revealed that the system had a positive impact on the key performance indicators for IDSR improved supportive supervision of the staff at operational levels including data transmission, and sharing of information for decision making. The innovation was well accepted by stakeholders and viewed by the frontline surveillance officers and health workers as a system that made reporting of IDSR data easy. The system if well harnessed will revolutionize the public health surveillance system in Nigeria.

## Supplementary Information

**Additional file 1.**

**Additional file 2.**

## Data Availability

The dataset used and analyzed during this study are available from the corresponding author on reasonable request.

## Abbreviations

DSNO: Disease surveillance and notification officer
eIDSR: Electronic Integrated Disease Surveillance and Response
ELR: Electronic Laboratory Reporting
EWARS: Early Warning Alert and Response System
FCT: Federal capital territory
IDSR: Integrated disease surveillance and response
HFs: Health facilities
ICT: Information communication technology
IT: Information technology
LGA: Local government area
LGA DSNO: Local government disease surveillance and notification officer
LGAF: Local government facilitators
mSERS: mobile Strengthening Emergency and Response System
MS: Microsoft
NCDC: Nigeria center for disease control
NHREC: National Health Research Ethics Committee of Nigeria
ODK: Open Data Kit
SMS: Short Message Services
SDSNO: State disease surveillance and notification officer
WHO: World health organization
