# Supplementary material for: Electronic reporting of integrated disease surveillance and response: lessons learned from northeast, Nigeria, 2019
Source: BMC Public Health. 2021 May 13;21:916. doi: 10.1186/s12889-021-10957-9 (PMC8117577; doi:10.1186/s12889-021-10957-9)
Supplement: Supplementary file 1 — Additional file 1. [file 12889_2021_10957_MOESM1_ESM.pdf]

eIDSR Evaluation, Health Facility Questionnaire

---

1) Date

2) What is your name?

3) What is your title?

4) What is your telephone number?

5) What is your email?

6) LGA Name

7) Health Facility Name

8) Health Facility Type

- Public
- Private
- Other - Write In:

9) What is the estimated catchment population of your health facility as of 2018?

10) Describe your understanding of eIDSR.

11) What is your role in eIDSR?

12) What phone do you use for eIDSR?

13) What else is the eIDSR phone used for?

14) In your view, was eIDSR well designed to suit your health facility?

- Yes
- No

15) Please explain your answer to the question above.

16) Is the eIDSR easy to use?

- Yes
- No
- Don't know - Write In (Required): ____________ *

17) Was there any alert detected in the last 3 months?

- **Yes**
- No

18) If you answered 'Yes' to the question above please describe it.

19) What was the time interval (in hours) between detection and notification of the last alert through eIDSR?

20) Did you use the data generated from eIDSR for action at this health facility?

- Yes
- No

21) Is your phone regularly charged?

- Yes
- No

22) Do you back up the data on your eIDSR phone?

- Yes

○ No

23) If you answered "Yes" please explain how you conduct the backup.

24) What is your source of charging your eIDSR mobile phone battery?

25) Do you always have airtime (credit) on the eIDSR phone?

○ Yes

○ No

26) Did you receive any training prior to the implementation of eIDSR?

○ Yes

○ No

27) Have you received a supervisory visit from the higher level since you began implementing eIDSR?

○ Yes

○ No

28) If “Yes” to the question above, how many supervisory visits have you received?

29) Do you find the eIDSR application simple to use?

○ Yes

○ No

30) Please explain your answer above.

31) Is there a paper-based back up for eIDSR?

- Yes
- No
- Other - Write In:

32) What value has eIDSR added to your surveillance system?

33) Has the eIDSR application ever crashed?

- Yes
- No

34) If you answered Yes to the question above, please explain what happened and how you resolved it.

35) Do you think eIDSR should be scaled up to the rest of the country?

- Yes

No

36) Please explain your answer above.

37) Does the eIDSR program add additional burden to your work?

Yes

No

38) If you answered Yes please explain your answer.

39) What were the major challenges in using eIDSR?

40) What would Nigeria lose if eIDSR was not scaled up?

41) Please provide any other comments on your experience with operating eIDSR.

Thank You!
