# Supplementary material for: Electronic reporting of integrated disease surveillance and response: lessons learned from northeast, Nigeria, 2019
Source: BMC Public Health. 2021 May 13;21:916. doi: 10.1186/s12889-021-10957-9 (PMC8117577; doi:10.1186/s12889-021-10957-9)
Supplement: Supplementary file 2 — Additional file 2. [file 12889_2021_10957_MOESM2_ESM.pdf]

Nigeria eIDSR Evaluation Stakeholders Questionnaire

---

1) Date

2) Name

3) Where do you work?

4) What is your position?

5) Email address

6) What are the major achievements of eIDSR in your view?

7) What were the major challenges that eIDSR is facing?

8) In your view, was eIDSR designed appropriately according to the Nigerian context?

- ○ Yes
- ○ No
- ○ I don't know

9) Please explain your answer above

10) Was eIDSR implemented appropriately according to the Nigerian context?

- ○ Yes
- ○ No
- ○ I don't know

11) Please explain your answer above

12) Does Nigeria find eIDSR useful?

- ○ Yes
- ○ No
- ○ I don't know

13) Please explain your answer above

14) Do you believe that the users of eIDSR find it simple to use?

- Yes
- No
- Other - Write In (Required): *

15) Do you believe that the users of eIDSR find it acceptable (meaning that all the intended users actually use it regularly)?

- Yes
- No
- Other - Write In (Required): *

16) Do you believe eIDSR is sensitive to identify public health problems at the health facility?

- Yes
- No
- Other - Write In (Required): *

17) Do you believe eIDSR provides adequate representation of all public health problems that occur at the health facility?

- Yes
- No
- Other - Write In (Required): *

18) Do you believe eIDSR provides timely notification of public health events at the health facility?

- Yes
- No
- Other - Write In (Required): ____ *

19) Do you believe eIDSR is a stable system (meaning that it doesn't crash and it is reliable)?

- Yes
- No
- Other - Write In (Required): ____ *

20) Do you believe that eIDSR provides quality data that can be used to make public health decisions?

- Yes
- No
- Other - Write In (Required): ____ *

21) Do you believe eIDSR is a flexible system (meaning that other conditions can be reported through it beyond those that are on the form)?

- Yes
- No
- Other - Write In (Required): ____ *

22) What is the extent to which eIDSR has adequately achieved its intended objectives?

23) Do you think eIDSR is a cost effective option for Nigeria?

- Yes
- No
- Other - Write In (Required): ____________ *

24) Please explain your answer above

25) What is the ability for the country to roll out eIDSR to all facilities, LGAs, and States?

26) To what extent did eIDSR contribute to public health surveillance and response in your state and Nigeria?

27) Please provide any other comments or suggestions to the evaluation team about eIDSR implementation and sustainability in Nigeria

Thank you so much for completing these questions fully. Please go back and fill out any remaining items if necessary before submitting.
